# Impact of tectonic and stratigraphic evolution of Shushan Basin on hydrocarbon accumulation, north Western Desert, Egypt

**DOI:** 10.1038/s41598-025-23532-8

**Published:** 2025-11-04

**Authors:** Mohamed Fagelnour, Sherif Farouk, Mohammad Abdelfattah Sarhan

**Affiliations:** 1 Geology Department, Faculty of Science, Helwan University, Cairo, Egypt; 2 Exploration Department, Egyptian Petroleum Research Institute, Nasr City, 11727 Egypt; 3 Geology Department, Faculty of Science, Damietta University, Damietta, 34517 Egypt

**Keywords:** Shushan Basin, Western Desert, Seismic interpretation, Structural modelling, Petrophysical analysis, Environmental sciences, Solid Earth sciences

## Abstract

Tectonic and stratigraphic evolution of the Shushan Basin in northern Western Desert of Egypt played an important role in hydrocarbon accumulation in the Mesozoic. The purpose of this study is to assess the hydrocarbon prospectivity of the deep Middle Jurassic Safa Member by integration of seismic interpretation, structural analysis, core descriptions, and wireline log evaluation over ten wells. Seismic data also indicate extensive E–W and NW–SE normal fault systems that developed horst–graben structures, hence providing crucial structural traps. Petrographic observation indicates that the Safa reservoir consists of vertically stacked, pebbly, cross-bedded sandstones deposited in a fluvial channel environment. Petrophysical evaluation indicates net pay thicknesses varying from 375 to 500 ft, 10–15% effective porosity, 5–20% shale content, and hydrocarbon saturation varying up to 95%, particularly in Qasr-3 and Qasr-4 wells. These results confirm the Safa Member as a fault compartmentalized, overlying Zahra shale-sealed, high-productivity gas–condensate reservoir. The findings also stress the significance of tectono-stratigraphic evolution in governing reservoir development and offer an exploration predictive model for such similar rift-related basins worldwide.

## Introduction

**Fig. 1:**
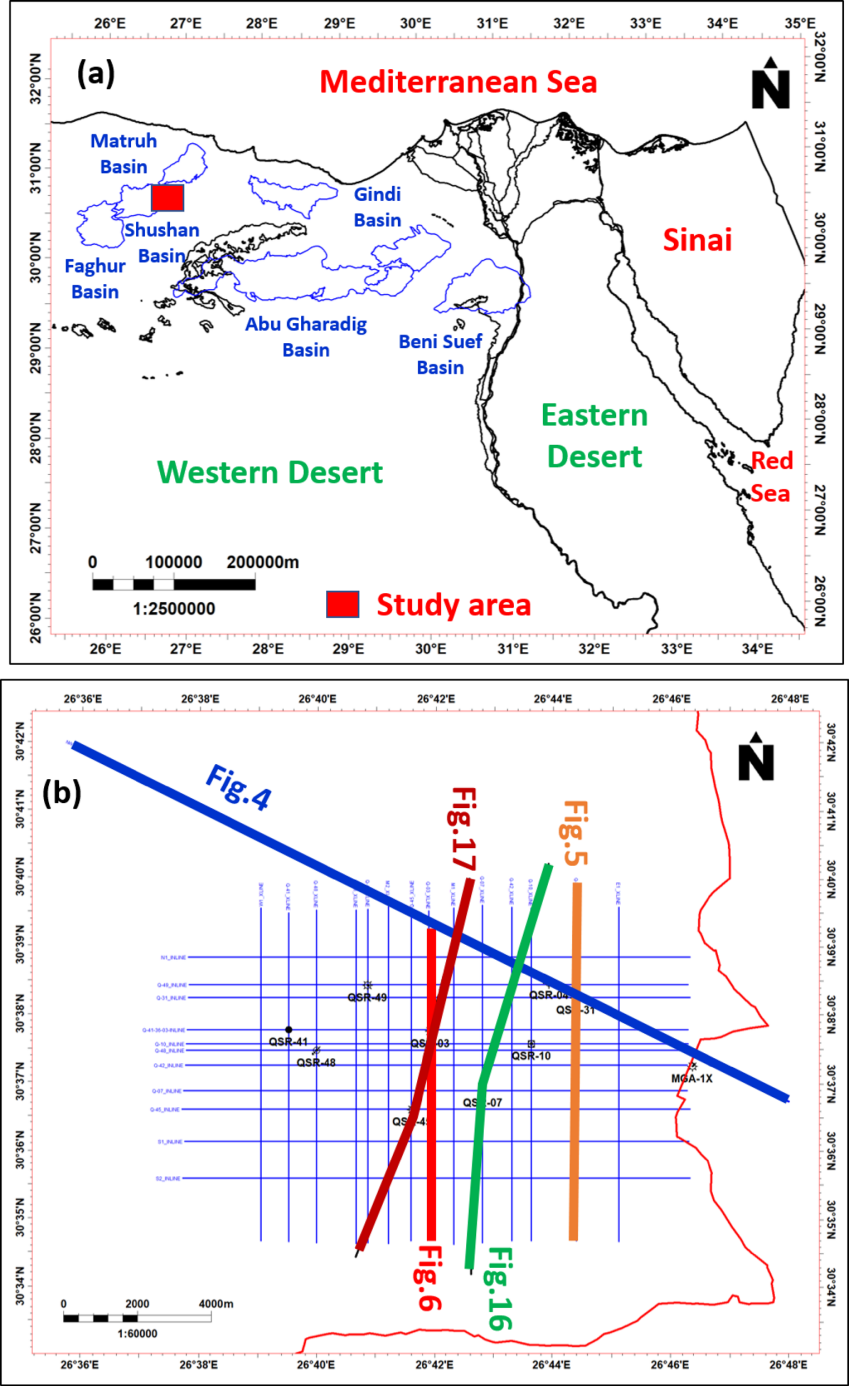
(**a**): Map represents the location of the study area within the Shushan Basin in north Western Desert. (**b**) Location map of wells, seismic lines, cross sections in the area of study. Petrel software, Version 2022. SLB. 11896.

The northwestern Egyptian Western Desert contains several rift-related sedimentary basins (Fig. 1a) that are North Africa’s principal hydrocarbon provinces^1,2^. Previous studies have demonstrated that these basins of long-standing exploration interest because of their tectonic evolution that created an array of structural traps, and widespread Mesozoic successions that hosted both prolific source rocks and clastic reservoirs^3–7^. In this regard, the Shushan Basin occupies a strategic position and is one of Egypt’s principal petroleum-producing areas^8–10^. Shushan Basin developed as a fault-controlled basin during Mesozoic rifting. The basin is bounded by NE–SW-trending faults later overprinted by NW–SE wrenching to produce tilted fault blocks, horsts, and half-graben structures^11,12^. These tectonic elements exerted strong control on sediment dispersal, accommodation space, and hydrocarbon entrapment. Notably, Jurassic–Cretaceous rifting controlled Khatatba Formation deposition, where Safa Member sandstones constitute one of the most significant reservoirs in the basin^13,14^. Despite this, previous research work has been directed towards highlighting the tectonic history^15–17^ or universal stratigraphic settings^18,19^, without incorporating structural interpretation, sedimentology, and petrophysical data extensively to determine the hydrocarbon potential of the Safa Member. The Safa Member is the lowest unit of Middle Jurassic Khatatba Formation and is composed mainly of fluvial sandstones interbedded shale and coal. The sandstone reservoirs, the carbonaceous coals and shales are source as well as seal facies and together form an entire petroleum system^20,21^. However, the distribution, quality, and the trapping mechanism of the Shushan Basin Safa reservoirs remain unconstrained to a significant extent, particularly with respect to syn-rift faulting and reservoir heterogeneity depositional controls. The area of study is situated in the southeastern part of Shushan Basin among latitudes, 30°34′, 30°42′N, & longitudes, 26°36′, 26°48′E (Fig. 1b). The study of subsurface structure and stratigraphy the Western Desert’s basins of Egypt and their impact on accumulation of hydrocarbons in Mesozoic reservoirs have been discussed by many authors^15–19^.

This study therefore integrates seismic interpretation, structural modeling, core description, and petrophysical analysis to evaluate the tectono-stratigraphic development and hydrocarbon potential of the Safa Member in the southeastern Shushan Basin. By integrating fault architecture with reservoir quality and distribution, the study completes knowledge gaps as well as underscores the control mechanisms for hydrocarbon accumulation in this deep reservoir.

The applicability of this study extends outside the study area. The Safa Member forms one of the main producing units of the other sedimentary basins located in the northern Western Desert, and its tectono-stratigraphic context contributes a predictive model to future exploration targets in this promising region.

## Geologic setting

The Shushan Basin is a Mesozoic extensional rift basin in northern Egyptian Western Desert with an intact thick sedimentary record from Paleozoic to Cenozoic^20,22^. It was Mesozoic extensional tectonic controlled, and NE–SW boundary faults were later overprinted by NW–SE wrenching. This structural regime produced horsts, half-grabens, and tilted fault blocks that governed accommodation space, sediment routing, and source and reservoir facies distribution^23,24^.

**Fig. 2:**
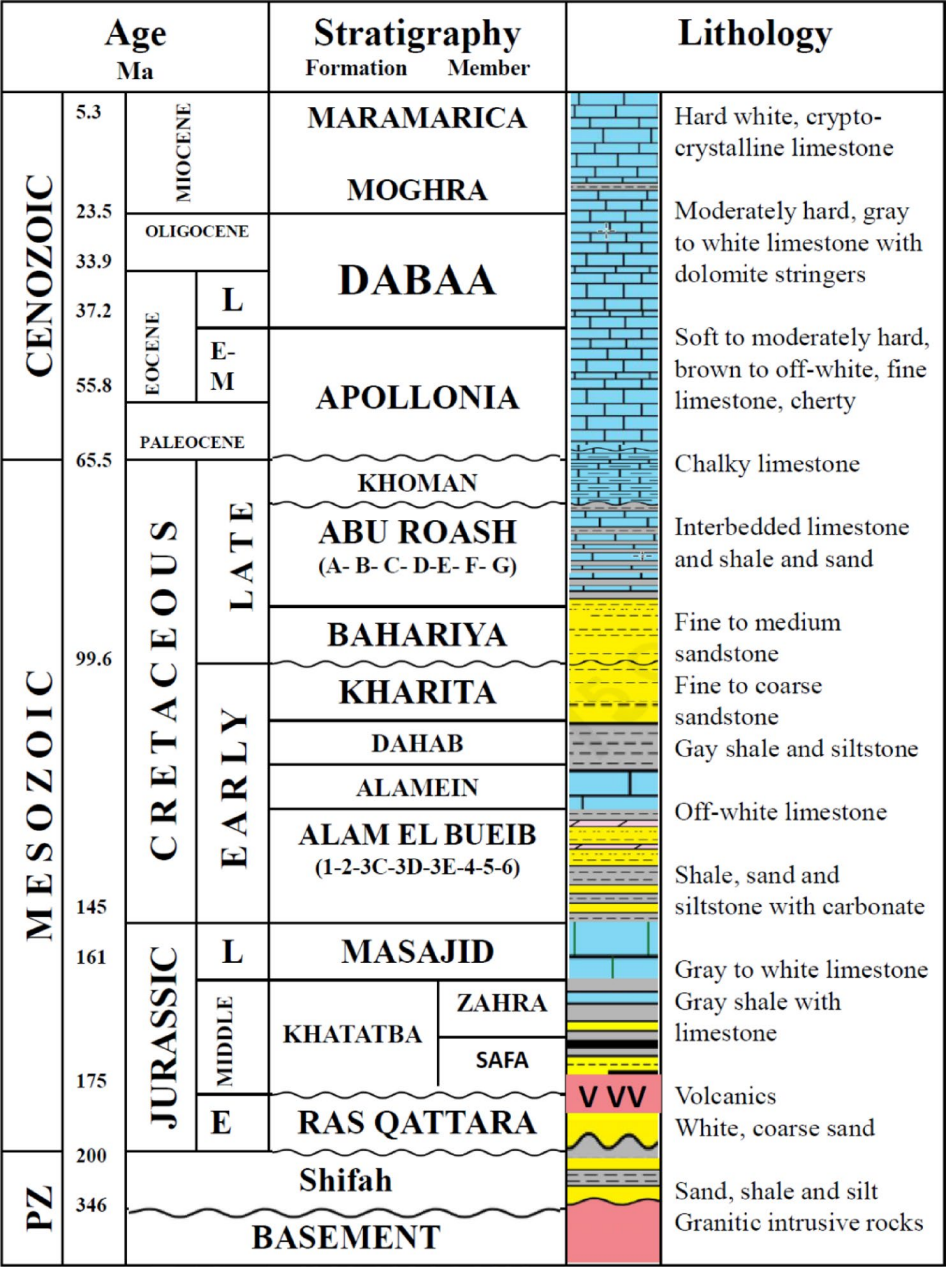
The stratigraphic column of the Western Desert Basins (after^15,23^).

Three main lithostratigraphic units (Fig. 2) were deposited in the Shushan Basin during the Jurassic: the Early Jurassic Ras Qattara Formation, the Middle Jurassic Khatatba Formation, and the Late Jurassic Masajid Formation^15,23^. The Ras Qattara Formation consists mostly of sandstones, locally capped with volcanic rocks related to episodic rifting and igneous activity^24,25^. It is overlain by the Khatatba Formation, which is further divided into the lower Safa Member and the upper Zahra Member. The Safa Member comprises interbedded marine sandstones and shale and coal, while the Zahra Member consists primarily of marine shales. The two units combined constitute a mature petroleum system, where the sandstones provide reservoirs, organic-rich shales and coals as source rocks, and the overlying shale sections as seals^21,26^. The Masajid Formation carbonates are an important Late Jurassic transgression of the sea, but they are eroded or absent in the uplifted southeastern margin of the basin^1^.

The Safa Member is particularly good as a deep gas–condensate reservoir. Its stacked channel sandstones within the fluvial environment are very prospective reservoirs, while interbedded shales and coals create both hydrocarbon generation and sealing^27,28^. Previous studies have identified the petroleum system elements of the Khatatba Formation^20,29^, but finer tectono-stratigraphic controls over Safa Member reservoir architecture within the Shushan Basin are poorly investigated. That is what this paper remedy through the integration of seismic, structural, sedimentological, and petrophysical observations to evaluate how tectonic evolution impacted reservoir distribution, quality, and trapping mechanisms.

## Data and methods

This study integrates well log data, seismic interpretation, core and petrographic observations, and structural modeling to evaluate the tectono-stratigraphic framework and hydrocarbon potential of the Safa Member in the Shushan Basin. The methodological workflow is outlined below.

### Well data

Wireline logs and mud logs from ten wells in the southeastern Shushan Basin (Fig. 1b) were analyzed. The log suite includes gamma ray (GR), neutron porosity, bulk density, photoelectric factor, sonic, and resistivity curves (microspherical, shallow, and deep). Shale content (Vsh) was estimated from GR logs^17,30^, effective porosity (PHIE) was calculated following standard equations^31^, and water saturation (Sw) was determined using the Archie equation^4. Hydrocarbon saturation (Sh) was derived as Sh = 1 − Sw. Cut-off values were applied to define pay zones: Sw ≤ 0.15, Sh ≥ 0.85, PHIE ≥ 0.12, and Vsh < 0.20.

### Seismic data

**Fig. 3:**
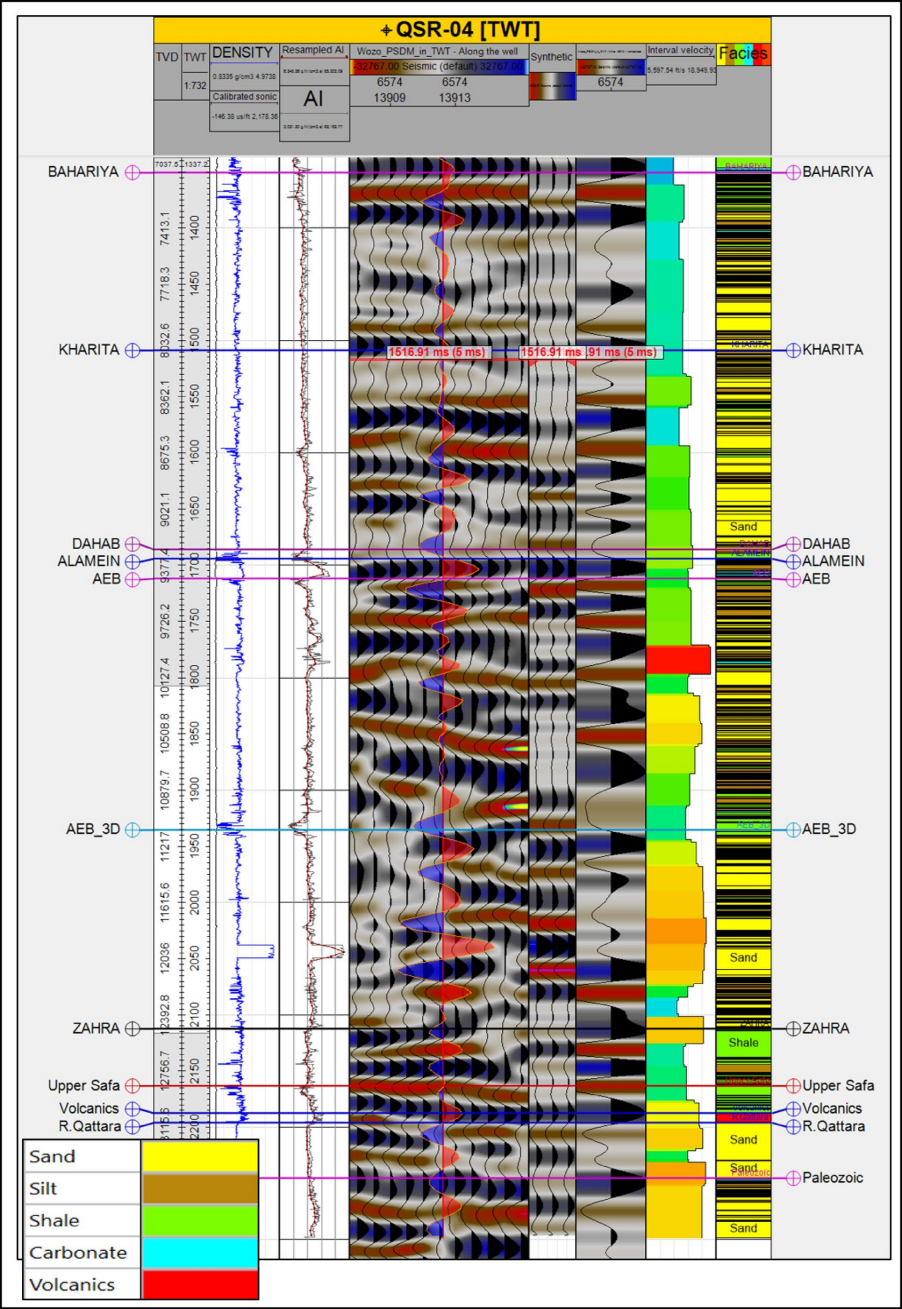
The synthetic seismogram generated from QSR-04 well. The Alamein Formation, marine-carbonate facies displayed higher-interval velocities and negative (red) amplitudes. The AEB-3D shale top shales possess low interval velocities and positive (blue) amplitudes. Zahra and the bottom Paleozoic shales are characterized by high interval velocity and positive seismic amplitudes.

A 3D post-stack seismic cube (zero phase, reverse polarity) with 26 vertical seismic sections^32^, covers the study area. Key stratigraphic strata such as the Bahariya Formation, Alam El Bueib Formation, Zahra Member, and Palaeozoic unconformity were the focus of seismic interpretation. Well tops were tied to seismic reflectors and synthetic seismograms were created using check-shot and sonic log data (Fig. 3). Using a linear velocity function^33^ and velocity modeling based on check-shot surveys, depth conversion was carried out.

### Core and petrography data

To determine lithofacies, depositional textures, and diagenetic characteristics, core slabs and thin sections from the Safa Member in the Qasr-3 well were analyzed. Grain size, sorting, roundness, and pore types were the main focus of the petrographic examination, which was conducted using Folk’s^34^ textural maturity scheme. Core laboratory measurements were used to calibrate the values of porosity and permeability.

### Structural modeling

To create structural models, Schlumberger PetrelTM software was used to import fault interpretations and seismic horizons. Two methods were used: (1) corner-point gridding for reservoir-scale representation, and (2) structural framework modeling to create horizon geometries^35^. Tilted blocks, horsts, and grabens were identified using fault sticks, which allowed for compartmentalization and structural trap analysis.

In order to determine how tectono-stratigraphic development regulates reservoir distribution and hydrocarbon accumulation in the Safa Member, this integrated approach combines petrophysical analysis with seismic and structural interpretation.

## Results

### Synthetic seismogram generation

A synthetic seismogram for the QSR-04 well was generated by merging calibrated sonic and density logs with check-shot survey data. The synthetic was matched to the seismic inline passing through the well (Fig. 3). The seismic cube-derived wavelet was zero-phase and reverse polarity, with troughs representing compressions. The match between synthetic and seismic reflectors was good, supporting horizon picks and permitting consistent correlation across the study area. Amplitude contrasts between shales, sandstones, and carbonates were clear and were used as markers for seismic interpretation.

In the Alamein Formation, marine-carbonate facies displayed higher-interval velocities and negative (red) amplitudes, under which were shales with positive (blue) amplitudes. Such a seismic response is also well represented on the synthetic seismogram of the QSR-04 well, which is tied to the seismic inline that intersects the well (Fig. 3). The AEB-3D shale top shales possess low interval velocities and positive (blue) amplitudes. Above these are the AEB-3D sandstone unit with slightly high interval velocities and distinct negative (red) seismic amplitudes. Especially high reflection coefficients were seen at the contacts between carbonates and clastics, and between sandstones and shales.

The thick Zahra shales and the bottom Paleozoic shales are characterized by high interval velocity and positive seismic amplitudes, which make them a good marker for picking in seismic horizon interpretation.

### Seismic interpretation

**Fig. 4:**
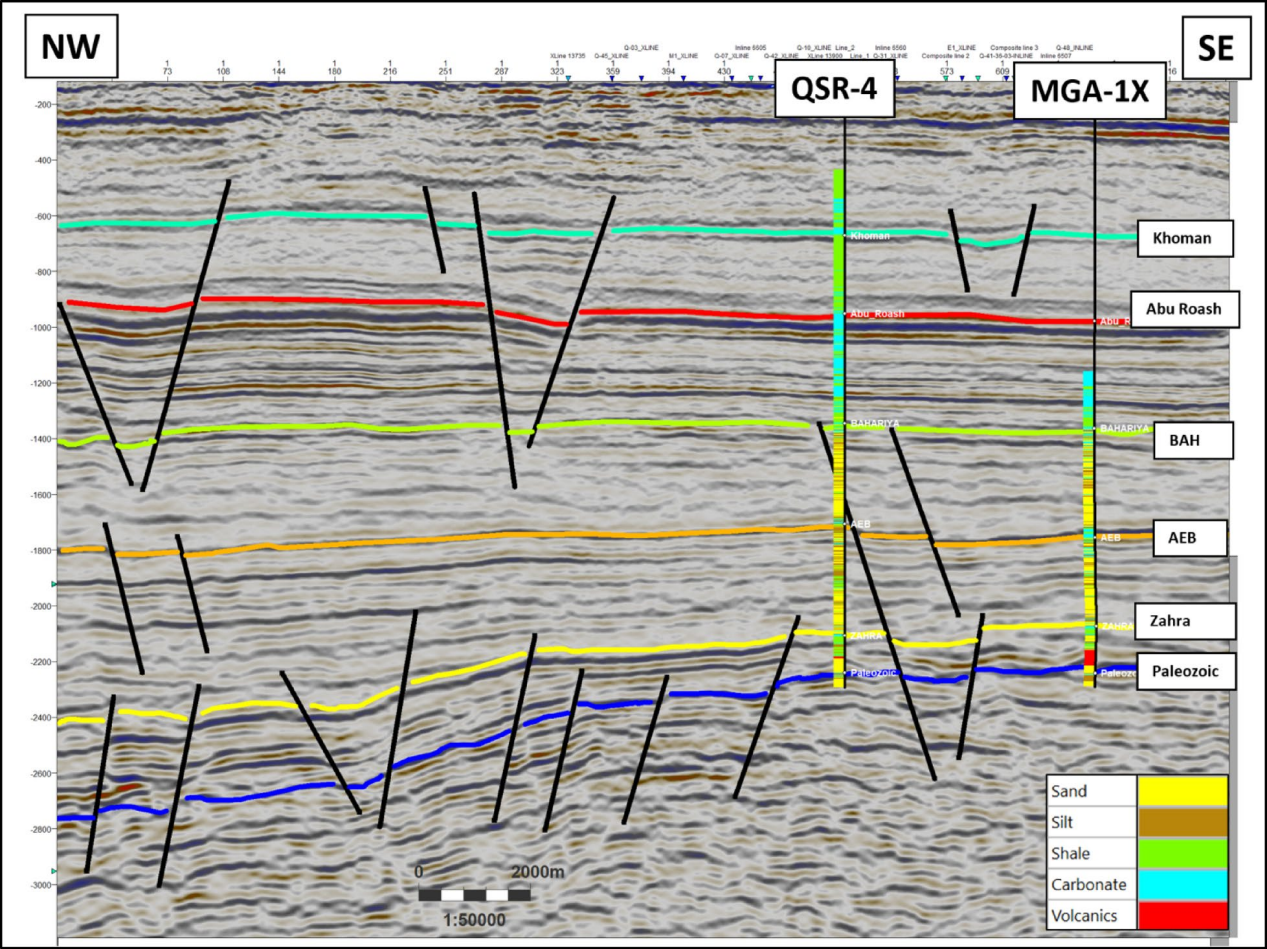
Interpreted NW–SE seismic line across the Shushan Basin (location in Fig. 1). The Jurassic (Zahra and Safa Members) and Lower Cretaceous (Alam El Bueib Formation) units thin towards the southeastern basin margin and thicken towards the depocenter in the northwest. Normal faults create tilted fault blocks that controlled accommodation space and reservoir distribution during syn-rift deposition. These structural geometries form the primary hydrocarbon traps of the study area.

**Fig. 5:**
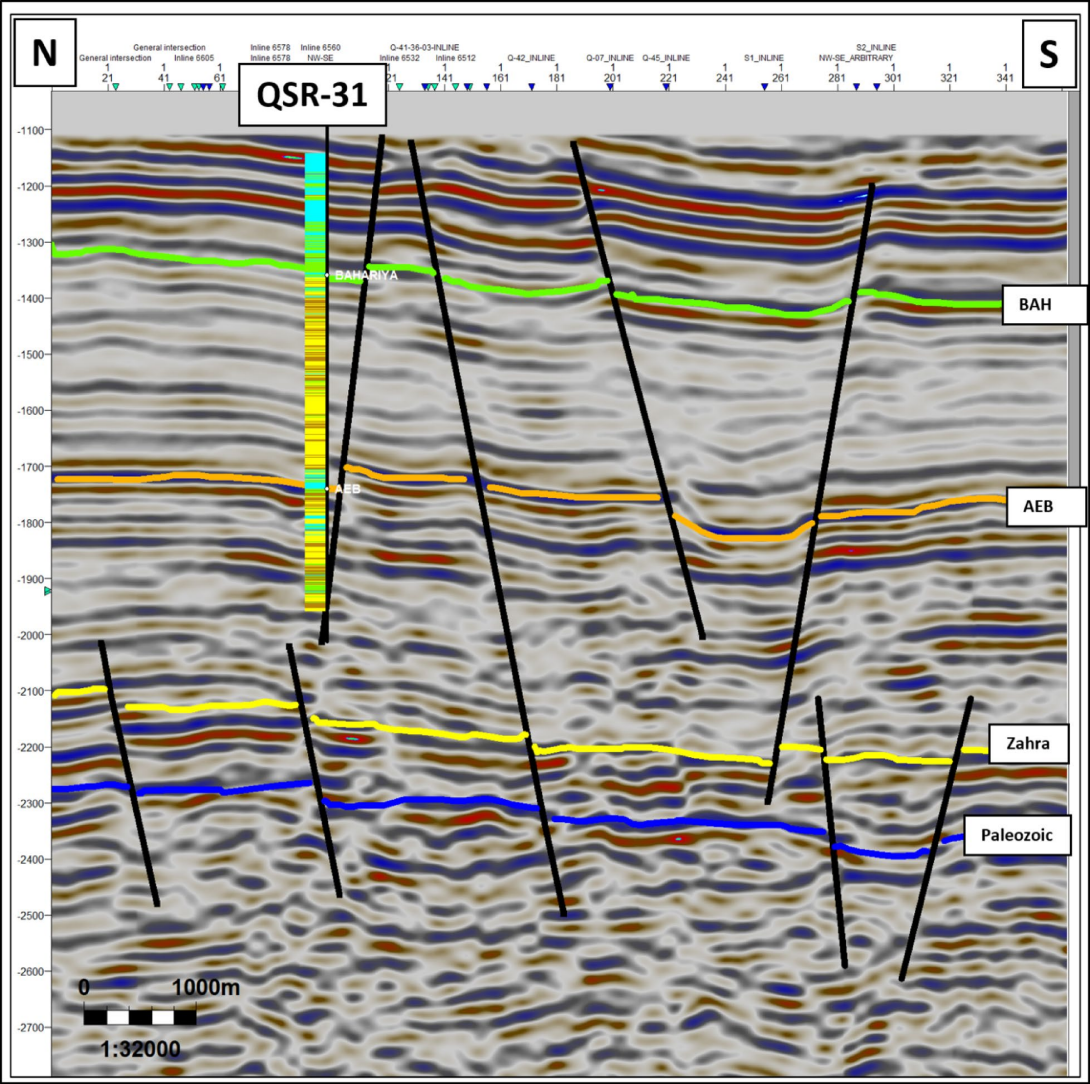
Interpreted N–S seismic section across the QSR-31 well (location in Fig. 1). The section shows multiple E–W and NW–SE trending normal faults that dissect the Jurassic and Cretaceous successions into tilted blocks. These horst–graben geometries illustrate the structural compartmentalization of the basin, which plays a key role in hydrocarbon entrapment within the Safa Member and overlying units.

**Fig. 6:**
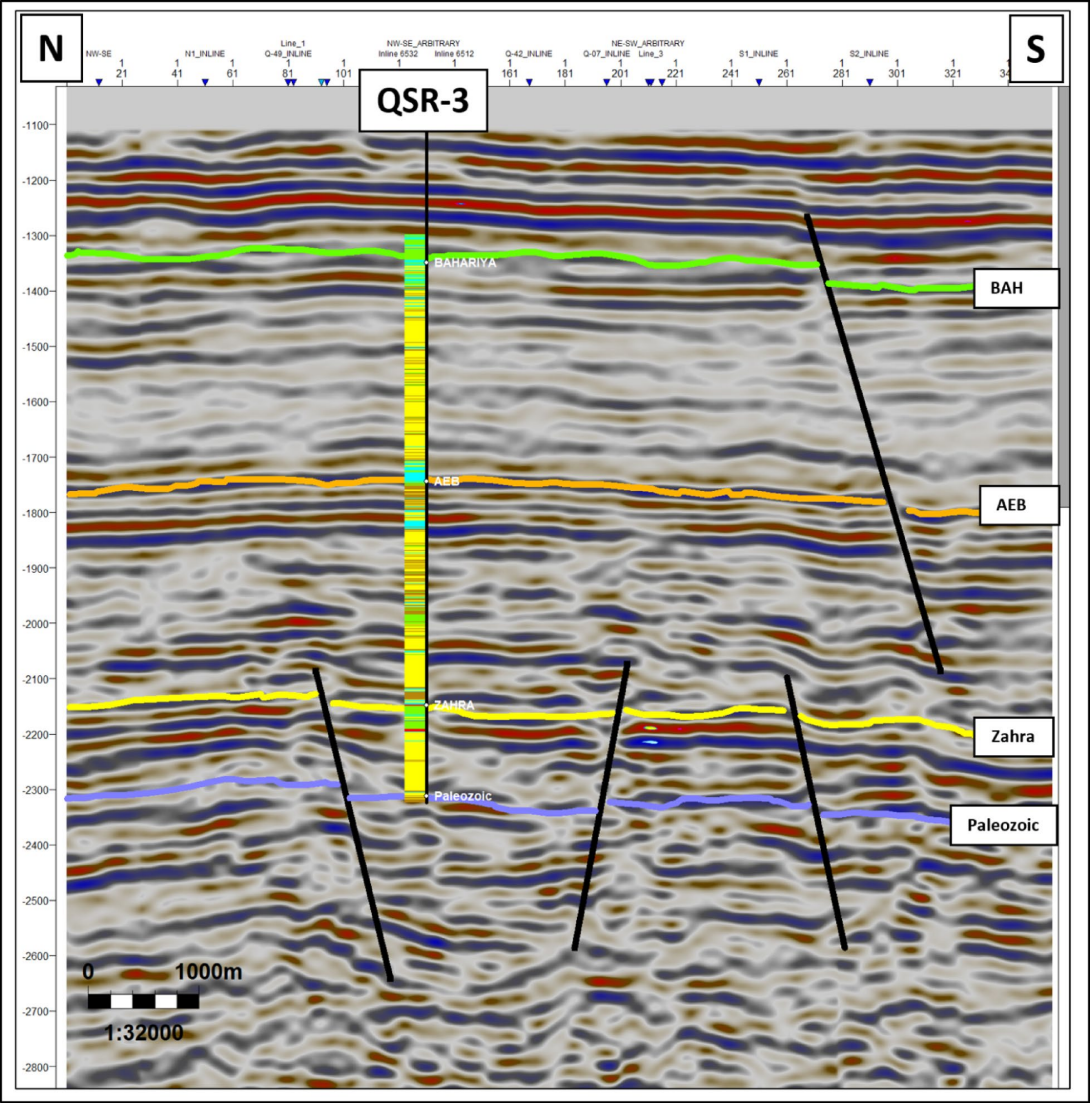
Interpreted N–S seismic section across the QSR-3 well (location in Fig. 1). The section reveals several normal faults affecting the Jurassic Zahra Member and Palaeozoic units, dividing them into tilted fault blocks. These structures demonstrate the horst–graben architecture typical of syn-rift settings and highlight the fault-bounded compartments that serve as key hydrocarbon traps in the Safa Member.

Four key stratigraphic horizons were mapped: The Upper Cretaceous Bahariya Formation, the Lower Cretaceous Alam El Bueib Formation, the Middle Jurassic Zahra Member, and the Palaeozoic unconformity. Interpretation recognized a set of E–W and NW–SE normal faults that divide the Jurassic and Cretaceous successions into tilted blocks (Figs. 4, 5, 6). Fault sticks were depth-converted by utilizing velocity models derived from check-shot data^33^. Regional seismic lines exhibit thinning of Jurassic and Lower Cretaceous units towards the basin margin and thickening towards the depocenter. Horst and graben geometries prevail in the subsurface architecture, creating multiple possible structural traps.

### Subsurface stratigraphy

**Fig. 7:**
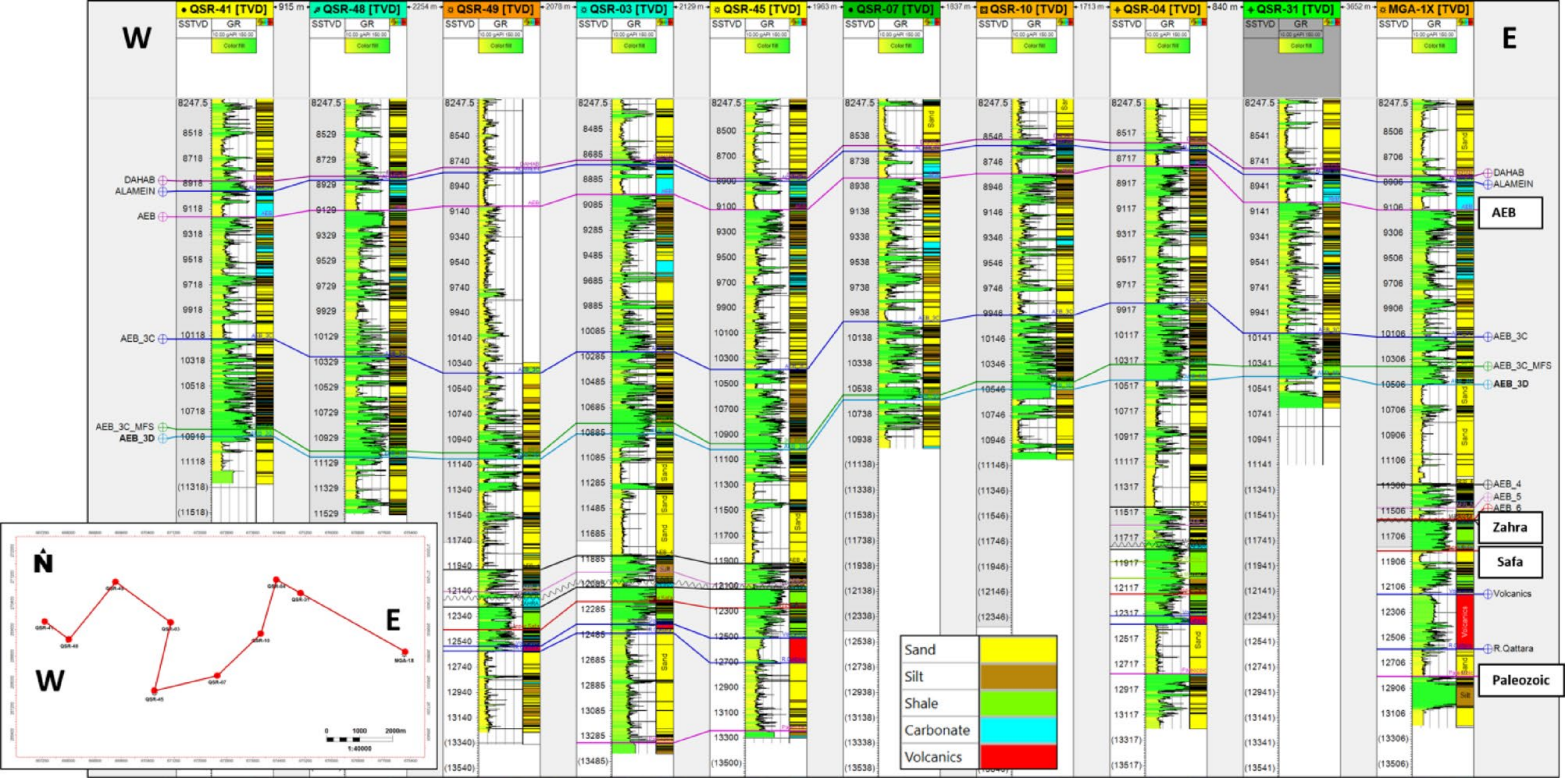
The East–West structural cross section shows the stratigraphic section from Palaeozoic to Dahab formations, and the Jurassic Khatatba (Zahra and Safa members), and Palaeozoic section through wells (QSR- 41, -48, -49, -03, -45-7, -10, -04, -31) and MGA-1X well.

**Fig. 8:**
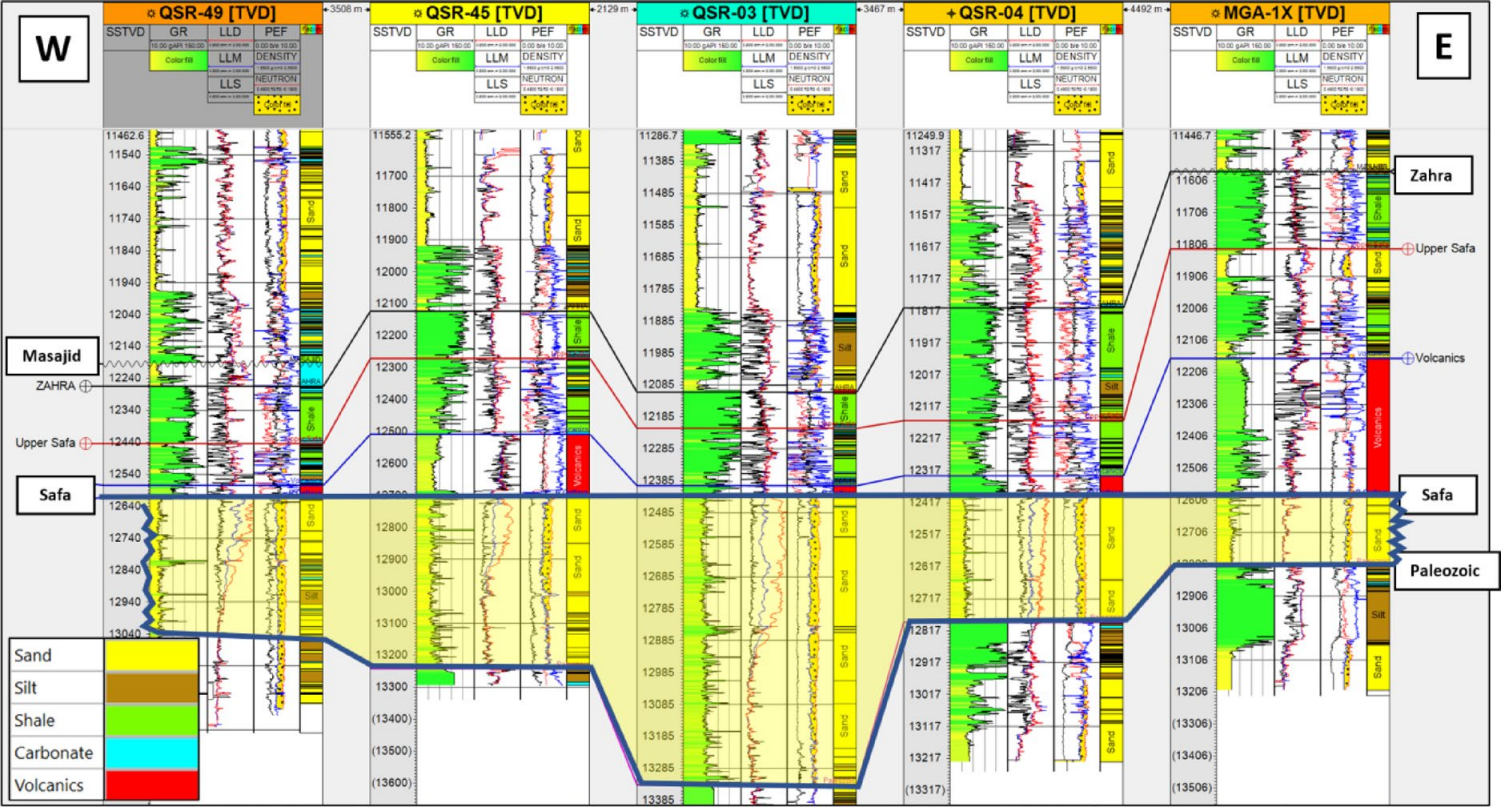
The E–W stratigraphic correlation flattened on top Safa Member. It shows that the maximum thickness of the sand channel is located at QSR-03 well and the thickness decreases towards east and west.

**Fig. 9:**
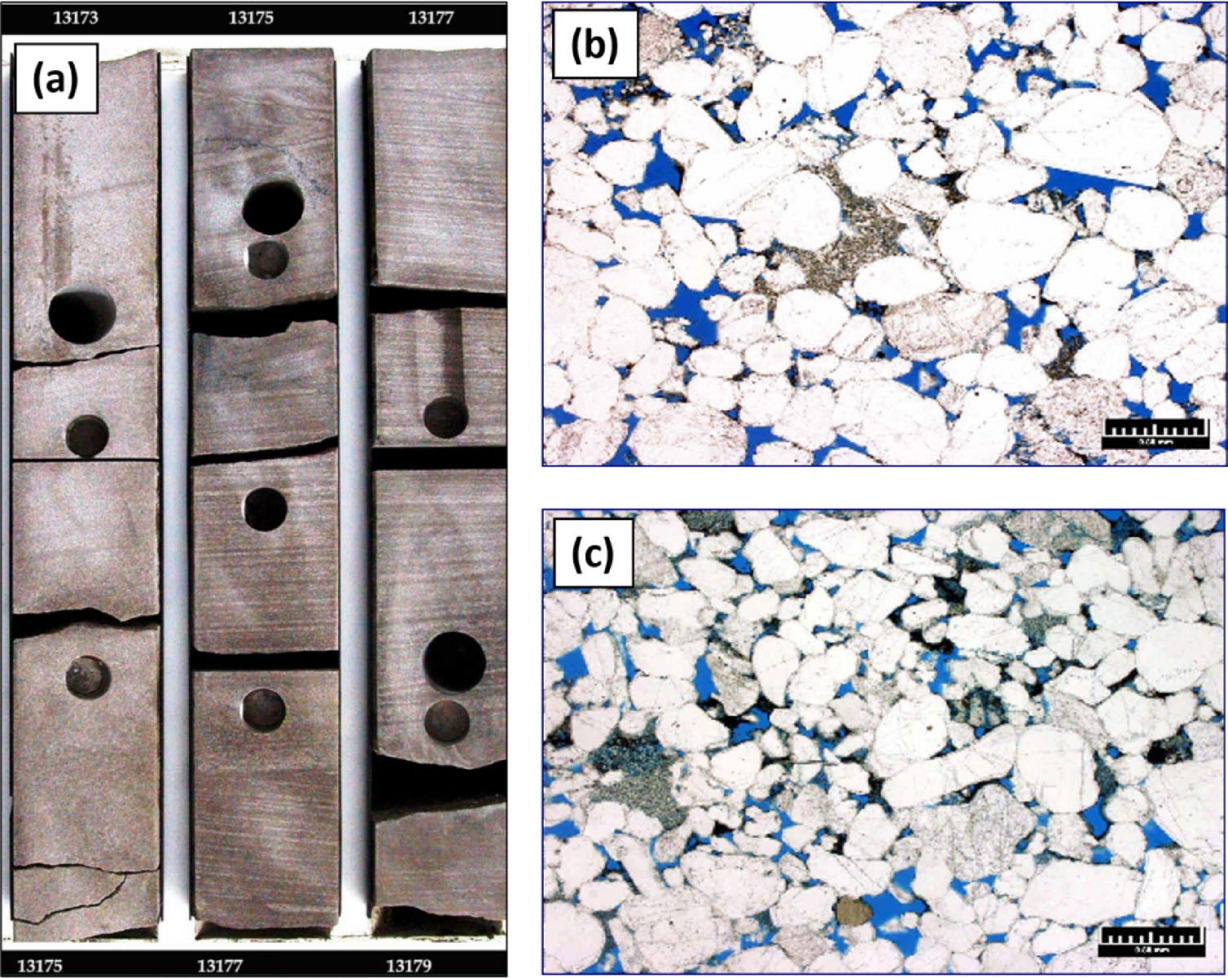
The core and section photos from QSR-03 well. (**a**) Photos pf slabbed core from 13,173 to 13,179 feet depth. (**b**) Thin section of plug sample at 13,166 depth shows coarse to medium grains, well to moderate sorting, rounded to sub-rounded grains (**c**) Thin section of plug sample at 13,178 depth shows very coarse-coarse to fine grains, bad sorting, subangular to angular grains. Thin section magnification: 25X.

**Fig. 10:**
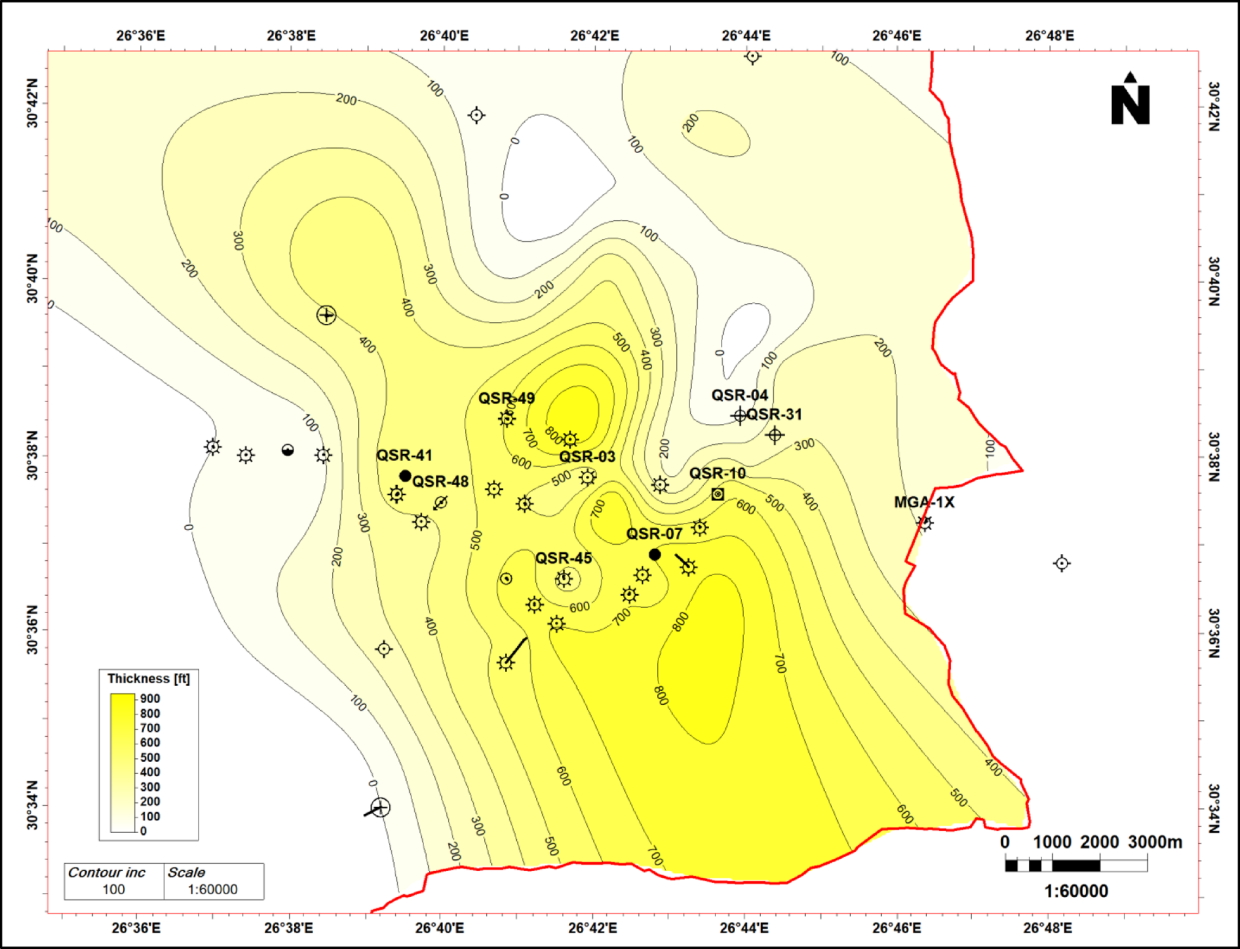
The net sand map of Safa Member, southeastern Shushan Basin. The basin edge is outlined by red solid line. The sand channel thickness ranges between 200 and 500 ft. and the Middle Jurassic Safa Member shows northwest to southeast trending sandstone channel.

The stratigraphic succession on Shushan Basin ranges in geologic age from Palaeozoic to recent, and the proved hydrocarbon reservoir potential is restricted mostly to Bahariya and Alam El Bueib formations, and Safa Member^1^. Qasr Field is selected in this study as an example of the giant gas and oil field located in southeastern Shushan Basin^17,18,32,36,37^. The study focuses on the Jurassic Safa sandstone as the primary gas and condensate reservoir, and the other reservoirs were discussed by many authors^32,38^**.** The East–West structural cross section passing through Qasr Field wells (QSR- 41, -48, -49, -03, -45-7, -10, -04, -31) and MGA-1X well show the penetrated section from Palaeozoic to Dahab formations (Fig. 7). Figure 7 shows the stratigraphic section from Palaeozoic to Dahab formations, and the Jurassic Khatatba (Zahra and Safa members), and Palaeozoic section are penetrated by QSR-49, -03, -45, -04, and MGA-1X wells only. The Palaeozoic section at bottom composed of siltstone and sandstone interbeds. It is overlain by the Early Jurassic Ras Qattara sandstone, which is overlain by volcanics and volcaniclastics related to the Jurassic rifting of Shushan Basin^24,25,35^. These volcanics are overlain by the Middle Jurassic Khatatba Formation which is divided into the Lower Safa sandstone Member, and the Upper Zahra shale Member. By the end of Jurassic, a big marine transgression led to the deposition of the Late Jurassic Masajid carbonate. The Masajid Fm. completely or partly eroded (or not-deposited) from southeastern Shushan Basin, where the area was mostly uplifted during this time^1^. The other wells, QSR-41, -48.- 07, -10, and QSR-31, are penetrating Alam El Bueib (AEB) Formation only at bottom. The AEB Formation were deposited in the Early-Cretaceous time and is classified into six members, which are composed of alternating sandstone, shale, and siltstone, representing fluviomarine depositional environment^15^. The E–W stratigraphic correlation flattened on top Safa Member shows that the maximum thickness of the sand channel is located at QSR-03 well and the thickness decreases towards east and west at MGA-1X and QSR-49 respectively (Fig. 8). The cored interval of the Jurassic Safa Member in QSR-3 well shows a vertically stacked succession of massive, light gray to gray, pebbly, cross- bedded sandstone, locally argillaceous (Fig. 9a). The thin section taken at 13,166 feet depth shows coarse to medium grains, well to moderate sorting, rounded to sub-rounded grains, with permeability 561 millidarcy and 10.9% porosity (Fig. 9b). The thin section taken at 13,178 feet depth shows very coarse-coarse to fine grains, bad sorting, subangular to angular grains, with permeability 142 millidarcy and 11.1% porosity (Fig. 9c). Petrographically, the quartz arenite is the main microfacies recorded within the studied samples, it represents 100% of the examined samples. The sandstones are generally well compacted, grins display long, occasionally concavo- convex contacts. These sediments were most likely formed in a high energy fluvial channel depositional context, as evidenced by the coarse grain size, poor sorting, and preponderance of tabular cross- bedding^34^. Mapping the average sand thickness in southeastern Shushan Basin, from wells’ data, shows that the sand channel thickness ranges between 200 and 500 ft. and the Middle Jurassic Safa Member shows northwest to southeast trending sandstone channel (Fig. 10).

### Petrophysical analysis

The examination of the mud logs in addition to the well logs data of the available four wells has confirmed that the sandstones of the Safa Member (the lower member of the Jurassic Khatatba Formation) exhibit favorable conditions to be considered as possible oil reservoirs in the two examined wells. The interpreted pay interval of QSR-4 well (Zone A) is located between depths; 13,100 to 13,475 ft., while, the deduced pay zone of QSR-3 well locates between depths; 13,150 to 13,650 ft. (Zone B). This conclusion is supported by a number of encouraging indicators, such as; the existence of oil stains and the higher values of the gas chromatographic analysis opposite several intervals within the Safa Member in the available mud logs.

**Fig. 11:**
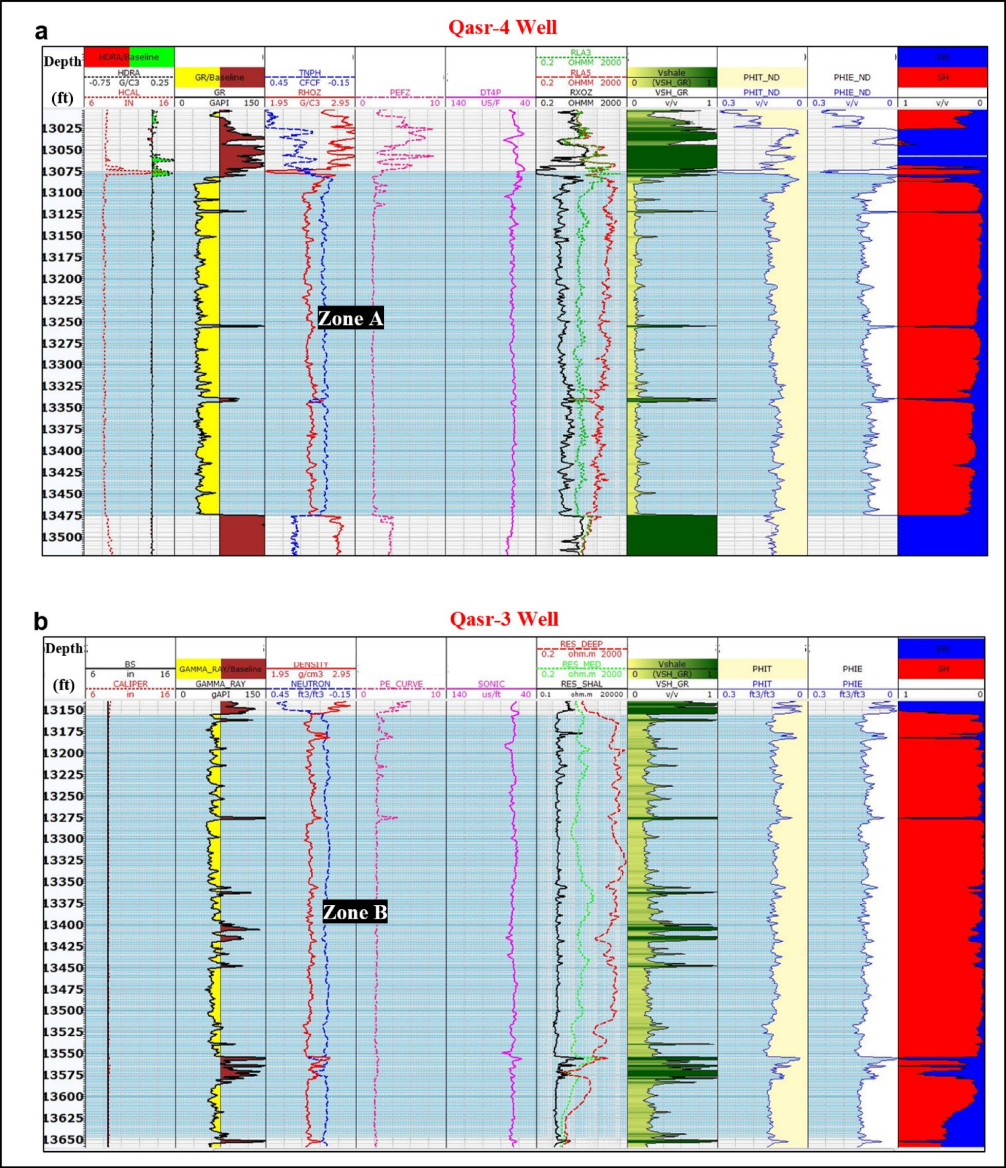
Petrophysical evaluation of the Safa Member in (**a**) QSR-4 and (**b**) QSR-3 wells. Both wells display clean sandstone intervals characterized by low gamma-ray readings, high deep resistivity values, and distinct neutron–density crossovers, indicating hydrocarbon-bearing zones. Zone A in QSR-4 (13,100–13,475 ft) and Zone B in QSR-3 (13,150–13,650 ft) show effective porosity between 0.12–0.16 and water saturation below 0.15, confirming their significance as productive reservoir intervals.

Analysis of the well log data indicates that these intervals are relatively clean, as evidenced by the low gamma ray readings, which suggest minimal shale content. The high values observed on the deep resistivity log reflect the likely presence of hydrocarbons, particularly oil. Additionally, elevated porosity readings from the neutron log further support the good reservoir quality of the targeted intervals. A characteristic cross-over between the density and neutron curves, typically indicative of a sandstone matrix was clearly observed within the interpreted pay zones (Fig. 11), reinforcing the identification of productive sandstone reservoirs.

**Fig. 12:**
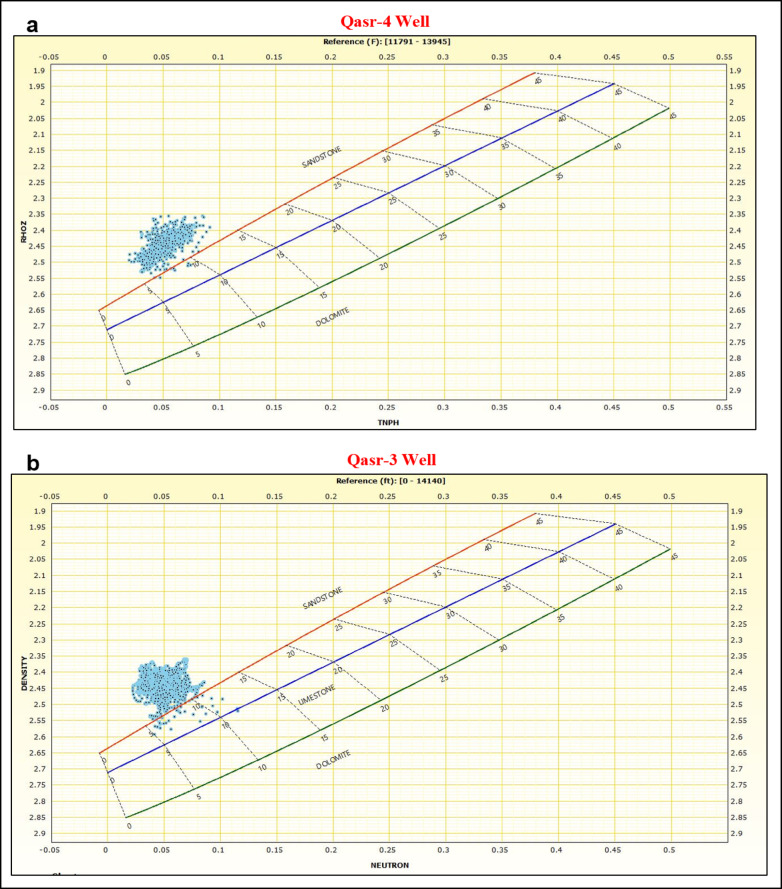
Neutron–density cross-plots of the interpreted pay zones in (**a**) QSR-4 and (**b**) QSR-3 wells. Most data points plot above the sandstone line, reflecting reduced bulk density caused by hydrocarbon presence. The clustering of values confirms the identification of productive sandstone intervals with good reservoir quality in both wells.

The created neutron-density cross-plots^39^ for Zones A and B show that the majority of the plotted points for each pay interval is clustering above the sandstone line confirming the relatively lower density caused by the presence of the hydrocarbon (Fig. 12).

**Fig. 13:**
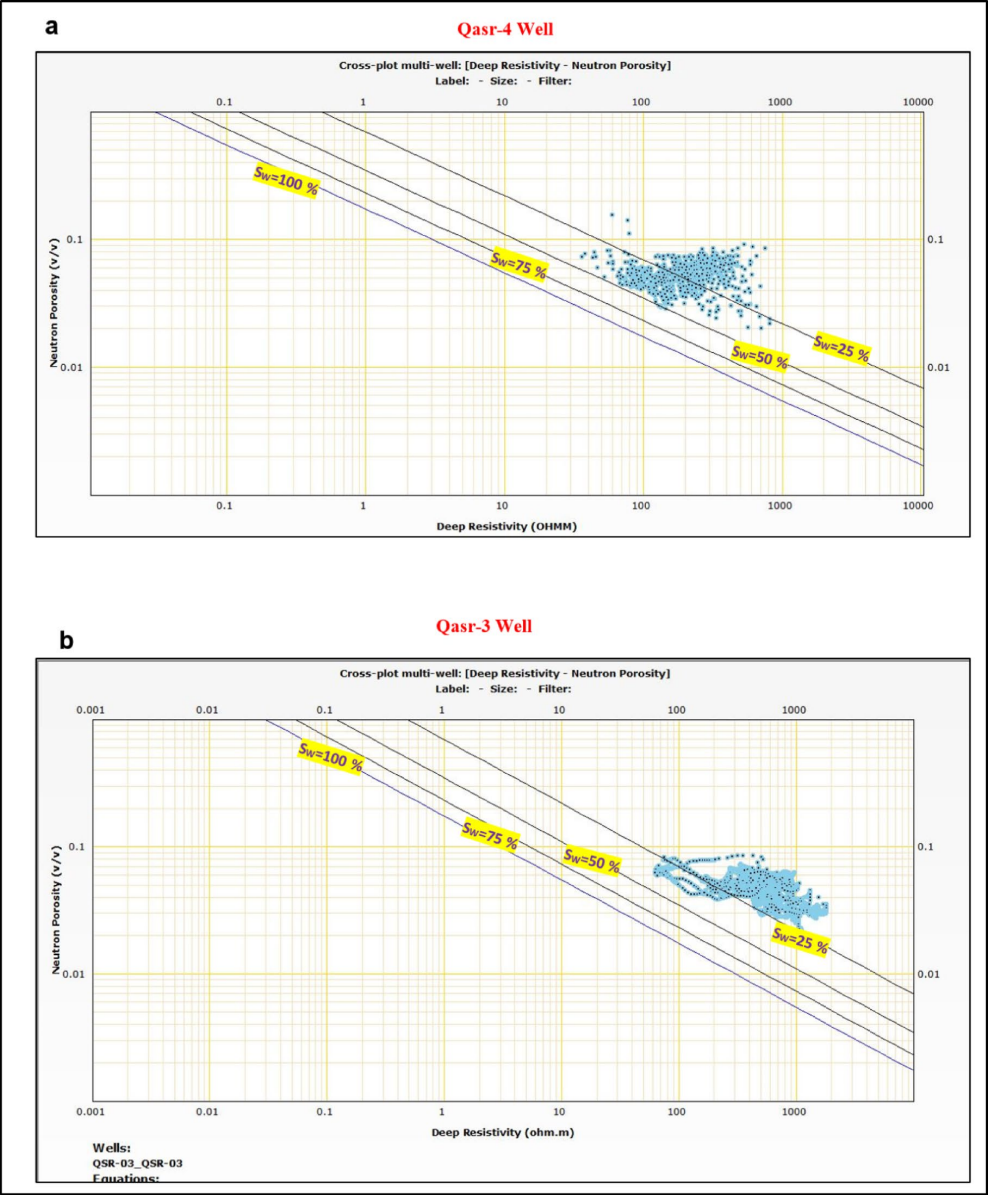
Pickett plots constructed for the interpreted pay zones in (**a**) QSR-4 and (**b**) QSR-3 wells. Most data points fall below the Sw = 50% line, supporting low water saturation and confirming hydrocarbon-bearing intervals. The alignment of data trends further validates the calculated petrophysical parameters and reinforces the reservoir potential of the Safa Member sandstones.

The applied cut-off values for identifying pay zones in the analyzed wells are as follows: water saturation ≤ 0.15, hydrocarbon saturation ≥ 0.85, effective porosity ≥ 0.12, and shale volume < 0.20. In Zone A of the QSR-4 well, the calculated petrophysical parameters show total porosity ranging from 0.13 to 0.16, effective-porosity between 0.12 and 0.15, shale volume between 0.05 and 0.10, and water saturation values ranging from 0.10 to 0.15, corresponding to hydrocarbon saturation values of 0.85 to 0.90 (Fig. 11a). In contrast, Zone B of the QSR-3 well exhibits total porosity values between 0.15 and 0.20, effective-porosity between 0.13 and 0.16, shale volume ranging from 0.15 to 0.20, and water saturation values between 0.05 and 0.10, indicating hydrocarbon saturation values between 0.90 and 0.95 (Fig. 11b). The constructed Pickett plots^35^ for Zones A and B show that most data points lie below the Sw = 50% line, further supporting the reliability of the calculated petrophysical parameters (Fig. 13).

### Structural analysis

**Fig. 14:**
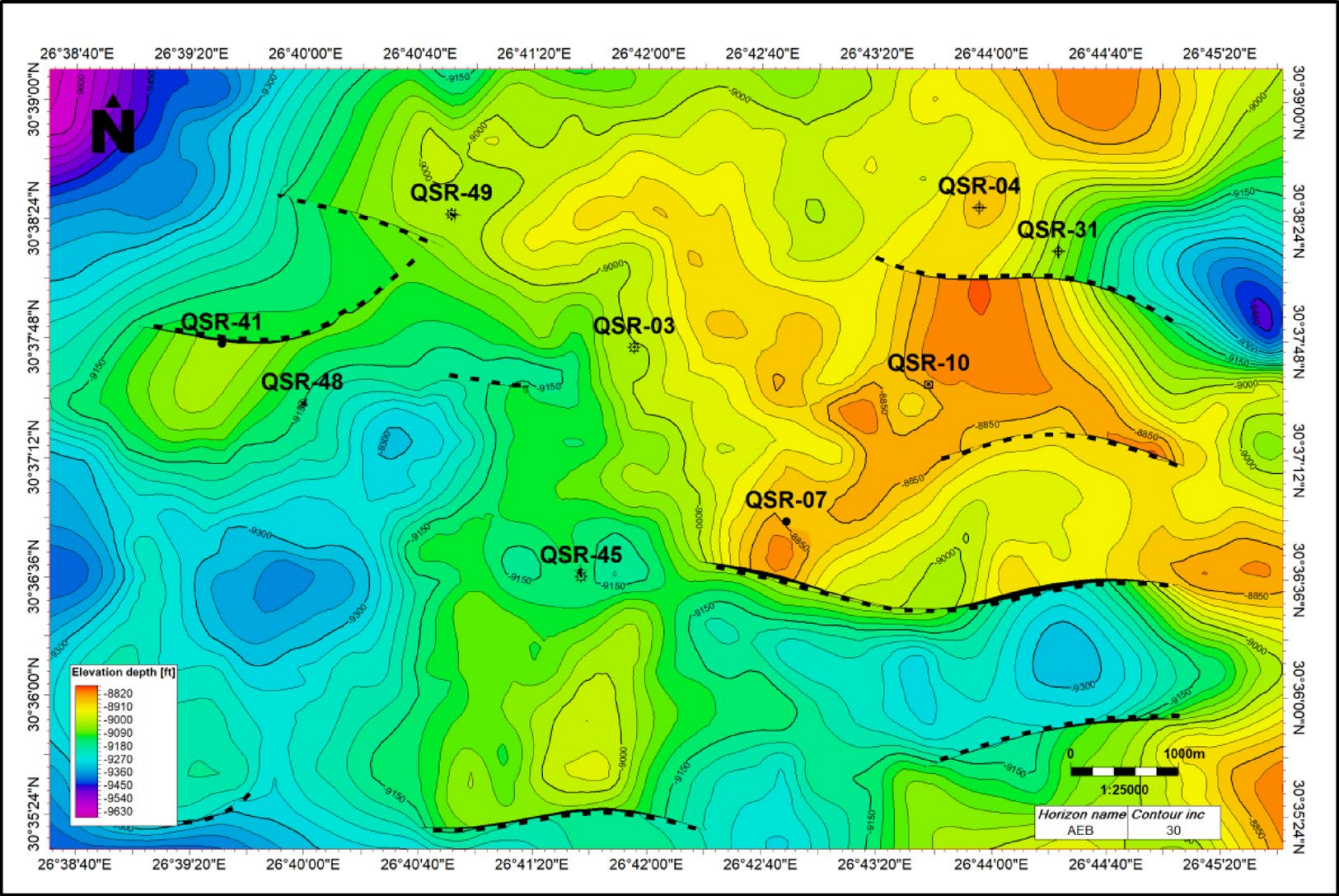
Alam El Bueib Formation depth structure map, measured in feet. It shows several tilted fault block structures bounded by E–W and ENE–WSW trending normal faults. Folds are observed along with faults, and described as faulted anticlines with a NE–SW trend.

**Fig. 15:**
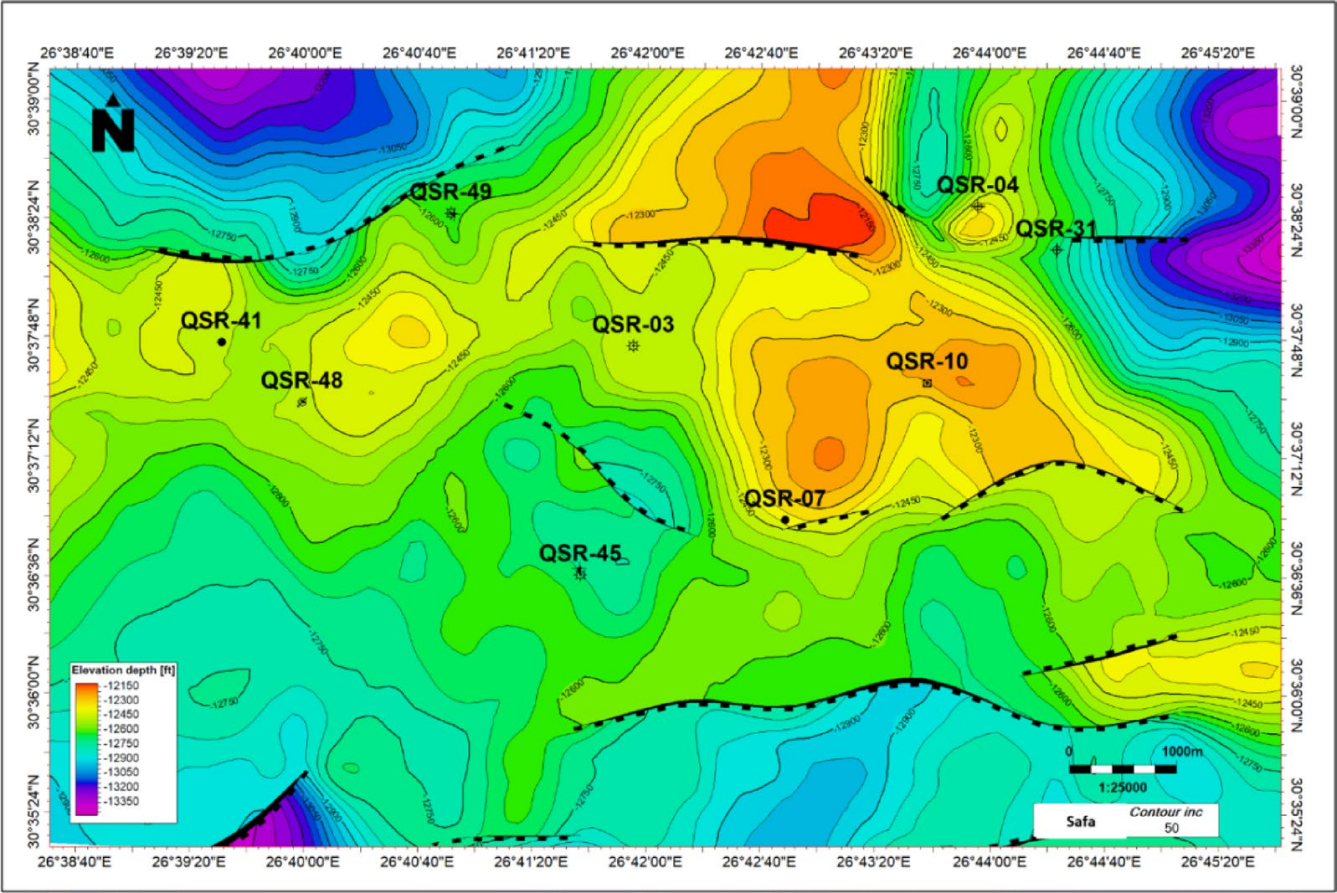
Safa Member depth structure map, measured in feet. It shows a number of tilted fault block structures bounded by E–W, ENE–WSW, and NW–SE trending normal faults. Folds are observed along with faults, either 4-way closures and/or faulted anticlines.

Grabens, half-grabens and normal faults that regulated the source and reservoir rocks deposition are typically found in the Jurassic-Cretaceous basins. The tectono-stratigraphic history, which produced a variety of reservoir-seal combinations, is intimately linked to the hydrocarbon potential in the north Western Desert^1^. The Lower Cretaceous Alam El-Bueib Formation depth structure map shows several tilted fault block structures bounded by E–W and ENE–WSW trending normal faults (Fig. 14). Several folds are observed along with faults, and described as faulted anticlines with a NE–SW trend. These structures form the main hydrocarbon traps of the Alam El Bueib sandstone reservoirs^17,18^. The Middle Jurassic Safa Member depth structure map shows a number of tilted fault block structures bounded by E–W, ENE–WSW, and NW–SE trending normal faults (Fig. 15). Several folds are observed along with faults, either 4-way closures and/or faulted anticlines. These structures form the main hydrocarbon traps of this deep sandstone reservoir.

## Discussion

The sandstones of the Khatatba Formation contain promising petrophysical characteristics for being potential hydrocarbon reservoirs^40^. The reservoirs quality, including porosity and permeability, are controlled by; the diagenetic changes, and the distribution of various cements, particularly carbonates and clay cements, and the depositional environment^41^. The Safa Member, the lowermost part of the Khatatba Formation, is primarily composed of sandstone intervals and a few thin, laterally continuous shale streaks^42,43^. The Safa Member in the Shushan Basin encompasses significant sandstone reservoir intervals that act as pay zones for hydrocarbon production. This study clarified the possibility that the Middle Jurassic Safa Member could serve as highly motivated gas and condensate sandstone reservoir in Shushan Basin. The seismic interpretation step, on the other hand, shows that there are many E–W and NW–SE striking normal faults. These faults might be suitable to serve as structural hydrocarbon traps in the region under study. The wire-line log data from tow drilled wells was qualitatively assessed, and the results showed that the Safa Member contain oil-bearing zone in QSR-4 well (Zone A) and QSR-3 well (Zone B). The quantitative evaluation of these reservoir intervals reveals that the net pay thickness ranges from 375 ft in Zone A to 500 ft in Zone B. Effective porosity varies between 0.12 and 0.16, shale volume ranges from 0.05 to 0.20, and water saturation lies between 0.05 and 0.15, corresponding to hydrocarbon saturation values of 0.85 to 0.95.

**Fig. 16:**
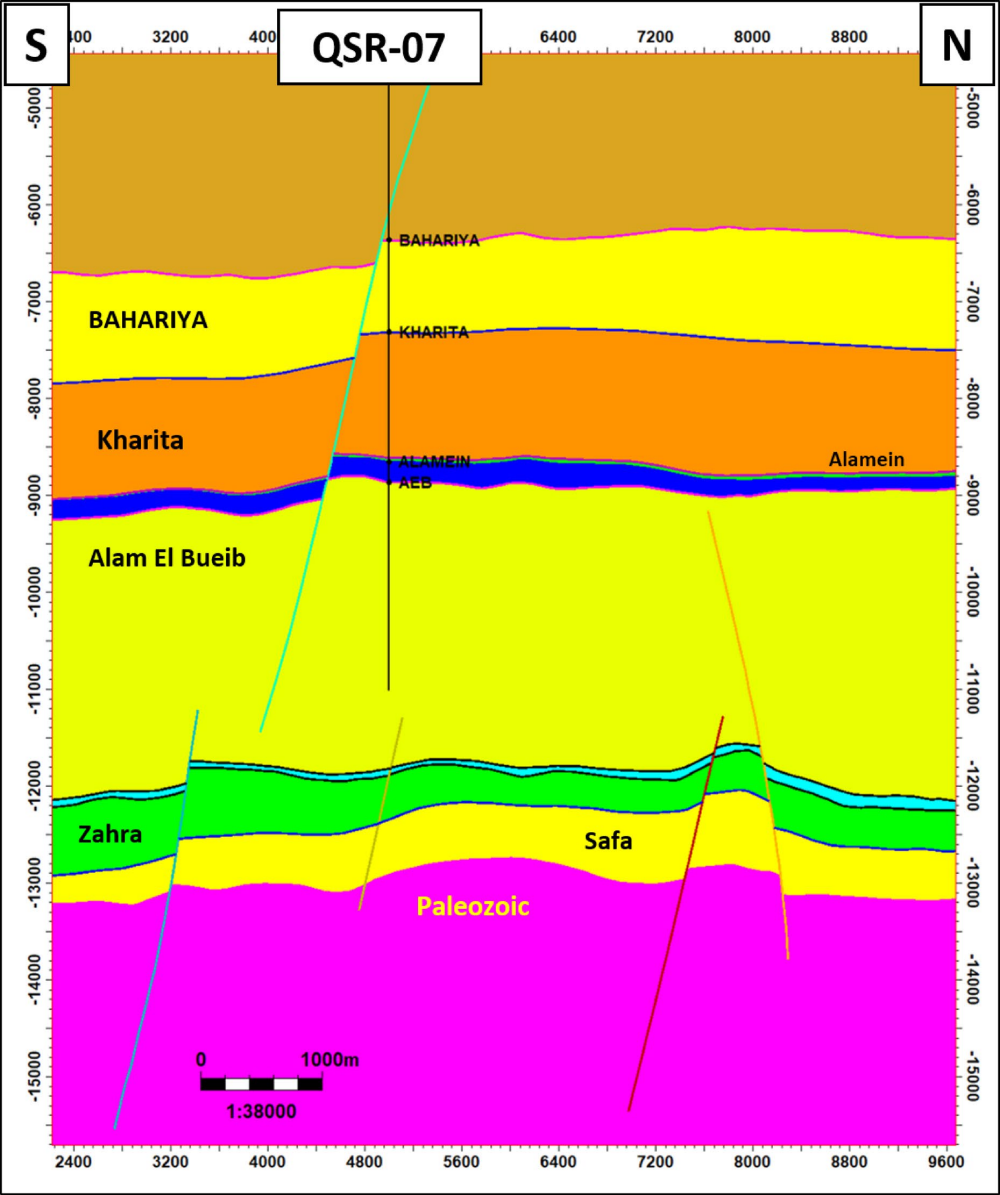
The north–south geologic cross section along QSR-07 well. (location in Fig. 1). It shows that the Middle Jurassic is highly dominated by normal faulting other than the shallower Alam El Bueib and Bahariya formations.

**Fig. 17:**
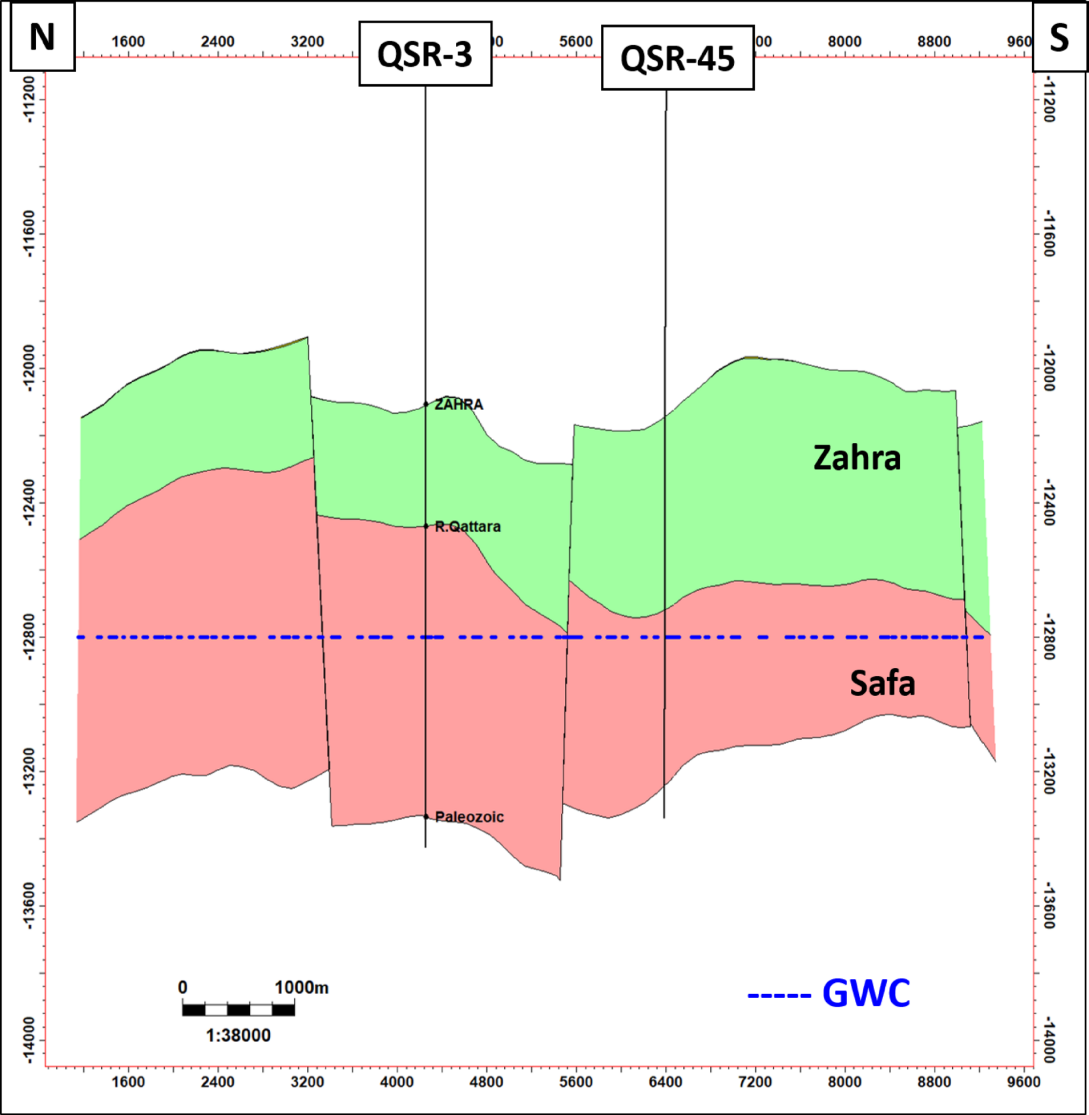
The north–south geologic cross section along QSR-3 and QSR-45 wells. (location in Fig. 1). It shows that Safa sandstone reservoir form several compartments bounded by normal faults.

Petrographically, all the analysed samples representing the reservoir interval in QSR-3 well (Zone B) can be classified into one microfacies (Quartz arenite) and are mineralogically mature. The grain size ranges from fine sand size and grade to very coarse sand size with maxima in coarse sand size fraction. The studied samples are moderately to well sorted and the grain roundness range from angular to rounded. In general, most of the examined samples are compacted, showing long contacts and close packing. The studied samples are texturally sub-mature. Porosity of the samples is dominated by primary intergranular pores with minor secondary inter- and intraparticle pores. The boundary faults of Shushan Basin follow a NE–SW trend in structure, which is counteracted by NW–SE later wrenching. It is primarily made up of elongated, parallel fault blocks that are tilted, or half-graben and horst structures, along with accompanying block erosion. The north south geologic cross section across QSR-07 well shows that the Middle Jurassic is highly dominated by normal faulting other than the shallower Alam El Bueib and Bahariya formations (Fig. 16). The N–S geologic cross section passing through QSR-45 and QSR-3 wells shows that Safa sandstone reservoir form several compartments bounded by normal faults (Fig. 17). These structural horsts and grabens series are the main traps of Safa deep sandstone reservoir in Shushan Basin.

From the integrated findings of this study, several broad geological and exploration strategic implications emerge of relevance not only to the Shushan Basin but also to basins of rifting origin in general. The spatial correlation between accommodation zones controlled by faults and the generation of high-quality fluvial sand bodies bears witness to syn-depositional tectonics in the control of sediment dispersal, reservoir thickness, and facies architecture. These trends are of worldwide importance, particularly in comparable Mesozoic rift basins in North and Central Africa (e.g., Sirte basin)^44^, where analogous structural and sedimentological drivers control reservoir formation. An awareness of these trends strengthens the need for predictive tectono-sedimentary models that integrate structural evolution with sediment input dynamics so that more targeted exploration can be conducted in underexplored frontier basins. Other global analogues include the basins of Europe, Western Black Sea, and the North Sea where the multiple Jurassic rifting and Late-Cretaceous- Early Cenozoic inversion created accommodation space for syn-rift sandstone reservoir deposition, and trap formation respectively^45,46^.

In heterogeneity setting of reservoirs, the Safa Member fluvial-derived has grain-scale heterogeneity, diagenetic change, and compartmentalization that all affect reservoir performance. These observations underscore the importance of employing the latest seismic geomorphology and inversion techniques spectral decomposition, coherency mapping, and neural-network-based characterization of facies to map subtle stratigraphic traps and improve resolution in the same type of stratigraphic conditions elsewhere worldwide. The effectiveness of these approaches has been demonstrated in both mature basins and new plays, which show broad applicability of the Shushan Basin model. Much like diagenetically controlled reservoirs of NW Borneo^47,48^, porosity of Safa Member is well-controlled by cementation and compaction, and affects the capacity for hydrocarbon storage. In reservoir development, the presence of fault-bounded compartments also emphasizes the necessity of dynamic modeling and pressure communication studies in fractured or segmented fluvial reservoirs. This combined approach combining static geological modeling with live production data, can be beneficial to global operators in the optimization of well placement tactics and recovery efficiency in complicated structural environments. Furthermore, the conclusions of this study enable a transition away from purely structure-based exploration towards process-oriented models focused on tectono-stratigraphic development and sediment routing systems. 

The results of this work offer a strong foundation for applied reservoir modeling, despite the fact that it concentrated on structural and petrophysical characterization. To estimate hydrocarbon amounts, assess reservoir connectivity, and improve field development tactics, the Petrel-based structural framework can be expanded to volumetric computations and dynamic simulations. Quantitative reservoir modeling that converts the geological characterisation described here into useful exploration and development results will be made possible by future work that incorporates production and pressure data. By using this integrated workflow to other complicated structurally basins around the world, this study contributes not just to regional understanding of the Shushan Basin but also to global best practices in hydrocarbon exploration and reservoir characterization in syn-rift and post-rift settings. Moreover, by offering the first comprehensive workflow that integrates seismic interpretation, structural modeling, core description, and petrophysical analysis to assess the Safa Member in the Shushan Basin, this study makes a novel contribution. Our method establishes a direct connection between syn-rift fault architecture and reservoir distribution, quality, and trapping efficiency, whereas previous research has focused on either tectonic history or reservoir parameters independently. This integration creates a prediction paradigm that can be used to other basins in northern Western Desert that are connected to the Jurassic rift, in addition to elucidating the controls on hydrocarbon accumulation in the Safa Member. The work enhances worldwide knowledge of syn-rift petroleum systems and regional exploration tactics by illustrating how tectono-stratigraphic development controls reservoir compartmentalization and hydrocarbon charge.

## Conclusions

- Integrated geophysical, sedimentological, and petrophysical interpretation confirms the Shushan Basin tectono-stratigraphic evolution to have dominated hydrocarbon trapping in the Safa Member. Jurassic–Cretaceous fault-bounded rifting horsts and grabens created effective structural traps that compartmentalized the reservoir and regulated hydrocarbon migration.
- The Safa Member has been established as a very productive gas–condensate reservoir, buried in stacked fluvial channel sandstones under syn-rift tectonic control. Petrographic and petrophysical information illustrates the interaction among depositional facies, diagenesis, and fault segmentation in the control of reservoir quality.
- Petrophysical interpretation of QSR-3 and QSR-4 wells confirms high-quality hydrocarbon-bearing zones. Effective porosity ranges from 12 to 16%, shale volume from 5 to 20%, and water saturation from 5 to 15%, providing hydrocarbon saturations of up to 95%. Net pay thickness is as much as 500 feet, indicating the reservoir’s enormous potential.
- Structurally, the Safa reservoir is compartmentalized by faulting, which aids trap integrity and is responsible for localized fluid accumulations. The interplay of Jurassic fluvial deposition, syn-rift faulting, and post-depositional tectonics has created an efficient petroleum system, with the Safa Member acting as a mature reservoir sealed by overlying Zahra Member shales.
- Aside from the local environment, the findings also indicate the significance of incorporating structural evolution into the sedimentary processes in rift basins. Seismic interpretation, structural modeling, core study, and petrophysical analysis as an integrated methodology offer a predictive approach towards discovering similar hydrocarbon plays in other Egyptian Jurassic basins.

## Data Availability

The data will be available from the corresponding author upon reasonable request and with permission of the Egyptian General Petroleum Corporation.
